# Nitrate capture and slow release in biochar amended compost and soil

**DOI:** 10.1371/journal.pone.0171214

**Published:** 2017-02-15

**Authors:** Nikolas Hagemann, Claudia I. Kammann, Hans-Peter Schmidt, Andreas Kappler, Sebastian Behrens

**Affiliations:** 1 Geomicrobiology, Center for Applied Geoscience, University of Tuebingen, Sigwartstrasse 10, Tuebingen, Germany; 2 Working group Climate Change Research for Special Crops, Department for Soil Science and Plant Nutrition, Hochschule Geisenheim University, Geisenheim, Germany; 3 Ithaka Institute for Carbon Strategies, Ancienne Eglise 9, Arbaz, Switzerland; 4 Department for Civil, Environmental, and Geo-Engineering, University of Minnesota, Minneapolis, MN, United States of America; 5 BioTechonology Institute, 140 Gortner Labs, 1479 Gortner Avenue, St. Paul, Mn, United States of America; RMIT University, AUSTRALIA

## Abstract

Slow release of nitrate by charred organic matter used as a soil amendment (i.e. biochar) was recently suggested as potential mechanism of nutrient delivery to plants which may explain some agronomic benefits of biochar. So far, isolated soil-aged and composted biochar particles were shown to release considerable amounts of nitrate only in extended (>1 h) extractions (“slow release”). In this study, we quantified nitrate and ammonium release by biochar-amended soil and compost during up to 167 h of repeated extractions in up to six consecutive steps to determine the effect of biochar on the overall mineral nitrogen retention. We used composts produced from mixed manures amended with three contrasting biochars prior to aerobic composting and a loamy soil that was amended with biochar three years prior to analysis and compared both to non-biochar amended controls. Composts were extracted with 2 M KCl at 22°C and 65°C, after sterilization, after treatment with H_2_O_2_, after removing biochar particles or without any modification. Soils were extracted with 2 M KCl at 22°C. Ammonium was continuously released during the extractions, independent of biochar amendment and is probably the result of abiotic ammonification. For the pure compost, nitrate extraction was complete after 1 h, while from biochar-amended composts, up to 30% of total nitrate extracted was only released during subsequent extraction steps. The loamy soil released 70% of its total nitrate amount in subsequent extractions, the biochar-amended soil 58%. However, biochar amendment doubled the amount of total extractable nitrate. Thus, biochar nitrate capture can be a relevant contribution to the overall nitrate retention in agroecosystems. Our results also indicate that the total nitrate amount in biochar amended soils and composts may frequently be underestimated. Furthermore, biochars could prevent nitrate loss from agroecosystems and may be developed into slow-release fertilizers to reduce global N fertilizer demands.

## Introduction

Biochar is defined as carbonized organic matter produced predominantly from agricultural residues [1] that can be applied in animal farming, manure treatment, as composting additive, and eventually as a soil amendment. Inspired by the global historic use of charcoal in agriculture [2–6], biochar is today mainly applied with the intention to increase crop yields. So far, this is done with mediocre success of just 18% grand mean yield increase across 60 studies around the world [7]. However, there are cases of up to three-[8] to fourfold crop yield increase [9], which need to be mechanistically understood to identify the factors that lead to plant growth promotion. The studies resulting in these remarkable yield increases both used low biochar application rates per hectare with high-dose root zone application together with a nitrogen source in planting basins. Biochar was either co-applied with inorganic NPKS fertilizer [8], or macerated in urine prior to co-application with compost [9].

The interaction of biochar with mineral and organic nitrogen species, specifically with nitrate, was recently suggested as one key mechanism of biochar plant growth promotion, as nitrate was shown to be slowly released from both soil-aged [10] and co-composted biochars [11]. The term “co-composted” refers to biochar which is mixed with compost feedstock (i.e. organic matter that is both rich in nutrients and labile organic carbon, e.g. manure) prior to aerobic composting [12]. This approach resulted in a compost of higher agronomic quality (superior plant growth promotion in pot trails) compared to the mixing of pristine/fresh biochar (no post-production treatment) into already matured compost [11].

Plant available nitrate in soil or compost is typically quantified by extraction with de-ionized water or 2 M KCl solution for 1 h [13]. Following up on the work of Kammann and colleagues [11], we define “slowly released nitrate” as nitrate that is only released in subsequent extractions, after an initial 1 h extraction. The underlying mechanisms of “nitrate slow release” are so far widely unknown and summarized as “nitrate capture”.

Slow release of nitrate by biochar might prevent nitrate leaching [10] and provide nitrate to plants over a longer period of time compared to non-biochar-amended composts or fertilized soils [11]. However, this effect has so far mostly described for isolated biochar particles, but has been hardly studied within the respective matrix, i.e. compost or soil. Thus, the relevance of slowly released nitrate by biochar can be questioned as also soil (clay, e.g. [14]) and compost might not release all nitrate and ammonium within 30 min or 1 h of extraction.

Therefore the goal of this study was to quantify the release of nitrate and ammonium from three different biochar-amended manure composts and a biochar-amended loamy soil in comparison to unamended compost and soil. Nitrate and ammonium were quantified following repeated extractions with 2 M KCl in 5 to 6 steps over a period of 1 week of total extraction time. We used different extraction conditions to gain further insights into the mechanisms of nitrate capture and slow release.

## Methods

### Biochar amended composts

Three different biochars were aerobically co-composted at 4.3% (w/w) in windrows [15] (20 m^3^ feedstock per treatment) with mixed manures and green plant material (manure: green plant material = 20:1 by weight) at the Ithaka Institute at St. Léonard, VS, Switzerland from August to October 2014 (biochar-amended composts CB1, CB2, CB3). In biochar-amended compost CB1, we used a mixed woody waste biochar B1 (700°C, Pyreg^®^ reactor [16]), in CB2 a sewage sludge char B2 (650°C, Pyreg^®^ reactor) and in CB3 a wood waste/pruning residue biochar B3 quenched with water (700°C, flame curtain pyrolysis in a KonTiki [17, 18]). Biochar properties were analyzed according to the requirements of European Biochar Certificate [1] by Eurofins Umwelt Ost GmbH, Halsbrücke-Tuttendorf, Germany and are listed in Table 1.

**Table 1:** Origin of biochars and characterization according to the requirements of the European Biochar Certificate.

	*EBC basic treshold*	*B1*	*B1 used in soil*	*B2*	*B3*
**Manufacturer**		SwissBiochar GmbH, Belmont-sur-Lausanne (VD), Switzerland	SwissBiochar GmbH, Belmont-sur-Lausanne (VD), Switzerland	Pyreg GmbH, Bingen. Germany	Ithaka institute, Arbaz (VS), Switzerland
***Technology***		Pyreg^®^	Pyreg^®^	Pyreg^®^	KonTiki–flame curtain pyrolysis
***Feedstock***		Mixed woody waste materials	Mixed woody waste materials	Sewage sludge	Vine wood
***HTT***^a^ ***[°C]***		700	700	650	750
***BET surface [m***^***2***^***g***^***-1***^***]***		200	232	60	252
***Ash 550°C [% w/w]***		18.2	19.4	76.5	18.4
***Elemental Composition***					
***H***		2.13	1.03	0.68	1.45
***C***	> 50	74.5	73.2	**21.3**	76.8
***N***		0.68	0.64	1.70	0.75
***O***		4.5	5.7	-2.7^c^	2.5
***S***		0.04	0.06	2.43	0.07
***Molar ratios***					
***H/C***	< 0.6	0.34	0.17	0.38	0.22
***H/C***_***org***_	< 0.7	0.34	0.18	0.38	0.23
***O/C***	< 0.4	0.045	0.058	-0.01	0.024
***pH***	< 10	8.3	8.0	7.2	8.5
***Salt [g kg***^***-1***^***]***		3.83	4.3	8.41	4.71
***Trace elements [mg kg***^***-1***^***]***					
***Pb***	< 150	3	< 2	27	4
***Cd***	<1.5	< 0.2	< 0.2	0.6	< 0.2
***Cu***	< 100	13	16	**610**	86
***Ni***	< 50	5	8	**65**	6
***Hg***	< 1	< 0.07	< 0.07	< 0.07	< 0.07
***Zn***	< 400	52	45	**1290**	200
***Cr***	< 90	6	10	**110**	9
***B***		24	36	74	41
***Mn***		190	310	1140	160
***Nutrients [mg kg***^***-1***^***]***					
***P***		870	1400	87000	2100
***Mg***		2700	3300	16000	4200
***Ca***		37000	49000	100000	40000
***K***		5800	8400	7200	4800
***Na***		740	830	6500	740
***Fe***		3900	2700	130000	3700
***Si***		28000	22000	36000	21000
***S***		230	400	230000	550
**PAH [mg kg**^**-1**^**]**^b^	12	4.60	6.70	2.40	6.90

Bold numbers indicate that EBC basic threshold was exceeded (in B2 only).

^a^ Highest Treatment Temperature

^b^ Sum of 16 polyaromatic hydrocarbons as suggested by the US Environmental Protection Agency (EPA), extracted with toluene as recommended by the EBC [1, 19]

^c^ Oxygen content is calculated after thermal oxidation as follows: [O] = 100% - [C] – [H] – [N] – [S] – [ash]. Negative values are the result of both CO_2_ precipitated as carbonate in the ash or reduced inorganic components of the biochar. The oxygen bound in the oxides of the inorganic compounds of the biochar increases the mass of the ash (pers. comm. Eurofins Umwelt Ost GmbH).

An additional control windrow (Con) was not amended with biochar. Frequent aeration (daily mechanical turning during the first three weeks, every three days thereafter) [15] resulted in composting temperatures of ~60°C for at least 2 weeks. After 63 days, composts contained <10 mg NH_4_^+^-N kg^-1^, had ambient temperature and were packaged in open plastic bags and stored frost-protected.

### Tübingen biochar field trial

In June 2012, B1 type biochar (same manufacturer, same type of feedstock, different batch, and separate analysis in Table 1) was incorporated at a rate of 60 Mg ha^-1^ into the upper 15 cm of a Terric Anthrosol (top- and subsoil of a Cambisol mixed by construction activities) at the Tübingen-Sand field site (lat. 48.5342, long. 9.0711). Before that, biochar was soaked in a diluted commercial fertilizer solution overnight (1:1 w/v, 1.25 g N kg^-1^ biochar) and control plots (no biochar amendment) received an equivalent amount of fertilizer. There was no further fertilization during the experiment, which included cropping with Emmer wheat (*Triticum dicoccon*) in 2012 and winter vetch (*Vicia villosa*) in 2013 and green fallow thereafter. Soil from biochar-amended plots and biochar-free control plots was sampled from the upper 15 cm in September 2015.

### Extraction procedures

Nitrate and ammonium were repeatedly extracted 1:10 (w/v) with 2 M KCl in 50 mL Falcon tubes in the dark on an end-to-end-shaker (HS501, IKA, Germany) at 150 rpm at room temperature (22±3°C), if not stated differently. After each step of extraction, Falcon tubes were centrifuged (4000 g, 10 min) and the supernatant was decanted into clean measuring cylinders to determine the volume of the decanted extractant. Aliquots were taken from the measuring cylinders for the quantification of nitrate and ammonium. Extractions were performed in triplicates. Composts without further modification were extracted both in February 2015, i.e. 6 months after beginning of the composting process, and in September/October/November 2015, i.e. after 12–14 months. After 6 months, composts were additionally extracted in a water bath at 70±1°C. After 12–14 months, composts were extracted additionally (i) after manually removing all biochar particles visible to the naked eye (“w/o biochar”), (ii) after γ-sterilization of the composts with 36.2 kGy by Synergy Health Allershausen GmbH, Germany, and extraction with autoclaved 2 M KCl under sterile conditions next to a Bunsen burner (“γ-sterilized”) and (iii) with a combined 2 M KCl + 3% H_2_O_2_ solution in the first step of extraction and 2 M KCl in the subsequent steps (“3% H_2_O_2_”). Biochar particles removed from the three biochar treatments were also analyzed and results were in line with previous studies [10, 11] showing slow release of nitrate by biochar. The results will be reported elsewhere [20].

### Nitrate and ammonium quantification and data analyses

Nitrate was quantified by continuous flow analysis (SEAL Analytical, Germany) after reduction to nitrite with hydrazine, which was prepared according to SEAL’s advice for soil extracts. Nitrite is quantified by UV-Vis absorption at 550 nm after reaction with N-1-naphtyl-ethylendiamin. Ammonium is quantified after reaction with sodium salicylate by UV-Vis absorption at 660 nm. The SEAL system is equipped with dialysis membrane, which removes any extraneous micro-particles to prevent side reactions or additional absorbance. We tested this system for artefacts due to the high content of dissolved organic carbon (DOC) and did not find any impact of DOC under relevant conditions. Detailed results are presented in the SI. Data were corrected for the residual extractant that could not be removed by decanting (residual volume of extractant = water content of sample + added extractant–decanted extractant) which carries a small amount of already extracted nitrogen from one to the subsequent extraction. Initial water content was determined after drying separate aliquots of composts and soils at 105°C for 48 h. Data were normalized to the dry matter content of the composts and presented as the average of triplicate extraction ± standard error. Statistical analysis was conducted in ORIGIN PRO 8 (OriginLab, Northampton, MA, USA). Differences between the different composts were tested with one-way-ANOVAs and Tukey test, differences between soil and biochar amended soil were assessed with the paired *t*-test. The original data are available through PANGAEA database (doi.pangaea.de/10.1594/PANGAEA.867498).

### Total nitrogen

Composts were freeze dried in March 2015, i.e. 7 months after setting up the composting windrows. After grinding in an agate mortar, samples were analyzed in triplicates in a CN element analyzer (vario EL, elementar, Hanau, Germany).

## Results & discussion

### Nitrate extraction at ambient temperature from compost after 6 months

**Fig 1:**
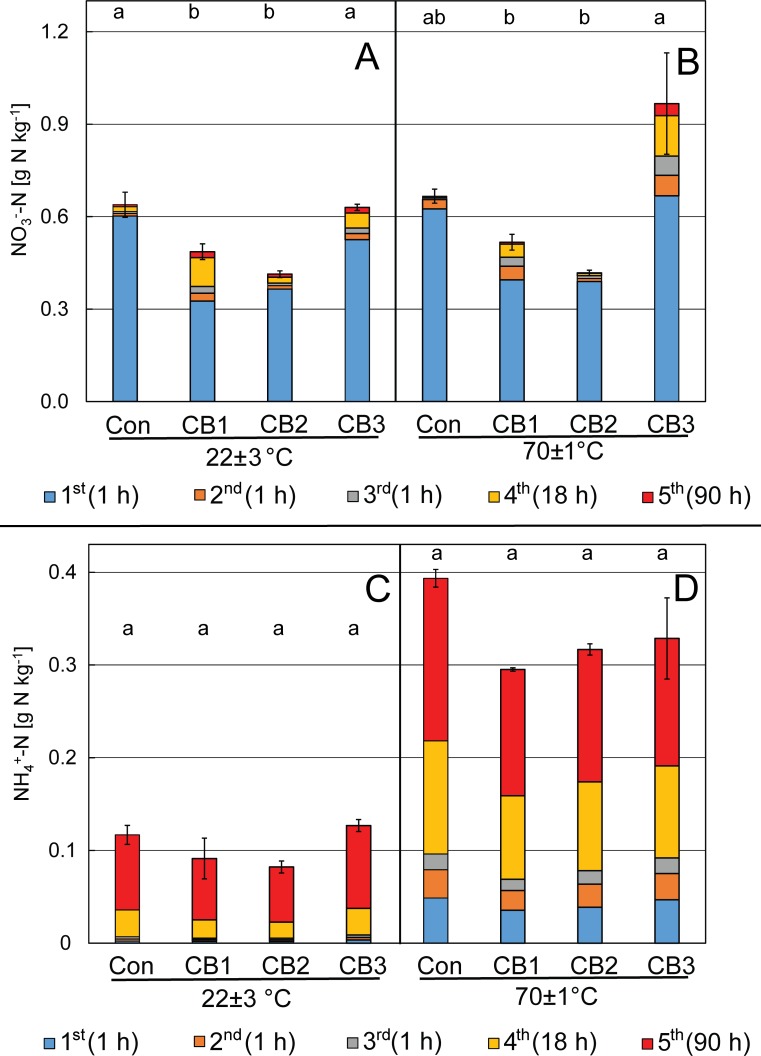
Mineral Nitrogen release during repeated extraction from 6 months old substrates. (A, B) Nitrate and (C, D) ammonium released from biochar-amended composts (Pyreg^®^—wood biochar amended compost CB1, Pyreg^®^—sewages sludge char amended compost CB2, Kon-Tiki–wood biochar amended compost CB3) and a non-amended control compost (Con) during consecutive, repeated extraction steps with 2 M KCl performed at ambient temperature on a shaker (“22±3°C”, A, C) or in a water bath (“70±1°C”, B, D). Extractions were performed 6 months after the beginning of the aerobic composting (2 months in aerobically managed windrows, 4 months storage in open plastic bags). Subsequent extraction steps with individual duration from 1 to 96 h are shown with increasing dark colors. Error bars indicate standard errors of triplicate extractions summated for 5 repeated extractions, lower case letters depict significant differences (0.05 level of an ANOVA) in total extracted amount of the respective N species with one set of extractions.

Typically, a 1 h extraction with e.g. 2 M KCl is used to quantify the amount of extractable NO_3_^-^ from soil or organic fertilizers such as compost [13]. After a total extraction time of 111 h (1 h + 1 h + 1 h + 18 h + 96 h), the amount of total extracted nitrate from the control compost did not differ considerably compared to the standard extraction procedure of 1 h only (0.64±0.07 vs. 0.60±0.06 g NO_3_^-^-N kg^-1^, [total extraction] vs. [1h extraction], Fig 1A). The extracts from biochar-amended composts showed just 54–87% of the nitrate content of the control compost according to the 1 h standard extraction procedure (Fig 1A). However, for all biochar-amended composts, additional NO_3_^-^ was released during the repeated extraction. Total NO_3_^-^ content increased by 52% (CB1), 14% (CB2) and 21% (CB3) after a total of 5 extractions steps compared to the nitrate content according to first 1 h of extraction of the respective sample. Only the biochar amended compost CB3 showed an extractable NO_3_^-^ content comparable to the non-biochar-amended control (0.63±0.07 g NO_3_), while the extractable NO_3_^-^ content of biochar-amended compost CB1 and sewage sludge char-amended compost CB2 was significantly lower, even after 1 week extraction time.

### Nitrate extraction at elevated temperature

In order to potentially increase extraction efficiency and to accelerate the extraction of nitrate, repeated extractions were conducted at 70±1°C. These extractions resulted in the same amounts of NO_3_^-^ released from non-biochar amended control compost, biochar amended compost CB1 and sewage sludge char amended compost CB2. However, a total of 0.97±0.21 g NO_3_^-^-N kg^-1^ were quantified by the extraction from CB3, i.e. 53% more compared to the extraction at 22±3°C on the shaker. This temperature-correlated increase of NO_3_^-^ was only quantified for CB3 and thus was caused by additional NO_3_^-^ release by biochar B3. B3, compared to B1 and B2, seems to have a greater capability to capture nitrate that is less accessible. Assuming that the difference of ~0.3 g N kg^-1^ between control compost and biochar-amended compost CB3 was solely released from the biochar contained in the CB3 substrate, the biochar B3 contains at least 3 g N kg^-1^, based on a final concentration of 10% biochar by weight. Initially, compost feedstock was amended with 4.3% biochar, but the composting process led to a mass loss due to oxidation of labile organic matter of the manure and thus to a relative increase of the biochar concentration to approximately 10% biochar [15].

### Biochar nitrate capture

The term “nitrate capture” refers to uptake mechanisms of nitrate by biochar which are not yet fully understood. Up to 5.3 g NO_3_^-^-N kg^-1^ was captured in co-composted biochar particles as described by Kammann and colleagues [11]. They used a different wood biochar produced at 600–700°C (Carbon Terra GmbH, Wallerstein, Germany), but the same composting protocol with slightly different feedstocks (additional use of rock powder, different composition of manures, extractions ~2–3 years after composting process). Thus, despite differences in the type of wood biochar, nitrate capture (3 vs. 5 g NO_3_^-^-N kg^-1^) was within the same range in our experiment. However, this capture of anionic nitrate by biochar cannot be fully explained by conventional anion exchange processes that are typically quantified as anion exchange capacity. Biochar is typically assumed to have a negative surface charge, which is responsible for its cation exchange capacity [21, 22]. Instead, non-conventional ion-water bonding/non-conventional hydrogen bonding have been suggested to be responsible for NO_3_^-^ retention by both co-composted and soil-aged biochar [11, 23, 24]. However, non-conventional hydrogen bonding on inner porous biochar surfaces have generally not been subject to systematic investigation. Comparing potential key properties of biochars such as BET specific surface area, content of major elements, trace metals and nutrient contents as well as H/C and O/C ratios (Table 1) did not reveal major differences between biochars B1 and B3, at least not on the bulk scale. However, B2, a sewage sludge char, had considerably lower carbon content, elevated trace metal content and a lower BET specific surface area than B1 and B3, which might explain the observed lower nitrate capture.

### Nitrate extraction after compost storage

**Fig 2:**
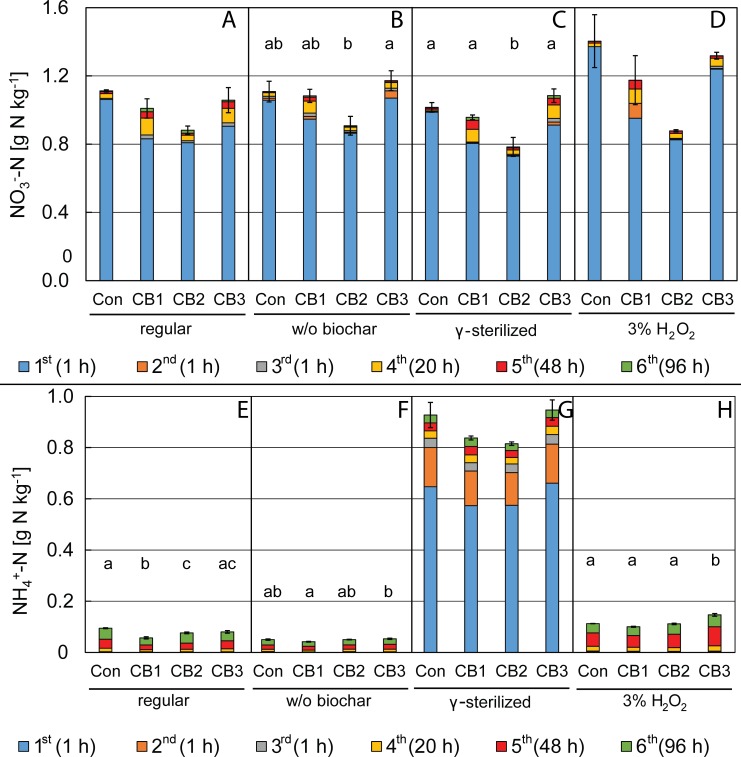
Mineral nitrogen release during repeated extraction from 12–14 months old biochar-amended composts. (A, B, C, D) Nitrate and (E, F, G, H) ammonium released from different biochar-amended composts (Pyreg^®^—wood biochar amended compost CB1, Pyreg^®^—sewages sludge char amended compost CB2, Kon-Tiki–wood biochar amended compost CB3) and a non-amended control compost (Con) during consecutive, repeated extraction steps with 2 M KCl (A, E) at ambient temperature on a shaker without further modification (“regular”), (B, F) after removing all biochar particles visible to the naked eye (“w/o biochar”), (C, G) after γ-sterilization of the composts (“γ-sterilized”) or (D, H) with a combined 2 M KCl + 3% H_2_O_2_ solution (“3% H_2_O_2_”) in the first extraction step. Extractions were performed 12 to 14 months after the beginning of the aerobic composting (2 months in aerobically managed windrows, subsequent storage storage in open plastic bags). Subsequent extraction steps with individual duration from 1 to 96 h are shown with increasing dark colors. Error bars indicate standard errors of triplicate extractions summated for 6 repeated extractions, lower case letters depict significant differences in total extracted amount of the respective N species with one set of extractions. If no lower case letters are shown, the average values were not significantly different by the 0.05 level of an ANOVA.

Extractions after 12–14 months of compost maturation resulted in considerable higher extractable NO_3_^-^ contents for all composts (Fig 2A) compared to the extraction after 6 months. Compost maturing leads to biomass mineralization; ammonification and nitrification are probably responsible for this increase in NO_3_^-^ [25]. This increase was quantified exclusively with the first extraction and was similar for all three biochar amended composts as well as the not-amended control. The absolute amount of NO_3_^-^ released from biochar amended composts in subsequent extractions (second to sixth extraction) did not differ from the results after 6 months. Thus, the relative contribution of slow nitrate release to the overall nitrate content of biochar-amended composts CB1, CB2 and CB3 decreased over time. Biochar was able to capture NO_3_^-^ released during the aerobic composting process in the windrows. After 2 months, the composting process was completed by conventional criteria (low NH_4_^+^ concentration, windrows cooled down to ambient temperature, very low CO_2_ production) and packed into open plastic bags for storage. During this storage, water content was lower than during composting in windrows (wet/dry ratio of compost: 1.7–1.8 vs. 1.8–2.1) and the composts were not mixed anymore by mechanical aeration. Thus, nitrate produced during storage might not be captured by biochar due to water transport limitations for the nitrate, i.e. nitrate might not have reached the biochar surfaces.

### Nitrate extraction from compost after removing biochar particles

After removing biochar particles, the effect of slow release of nitrate in subsequent extractions disappeared for biochar-amended compost CB2 (amount of slowly released nitrate < standard error of all extractions) (Fig 2B). B2 had a granular structure which was easy to manually separate from the compost matrix. Biochar-amended composts CB1 and CB3 still showed slow release of nitrate even after removing all biochar particles visible to the naked eye, but to a lower extent (66–76% of slowly released nitrate in composts prior to biochar removal). Most likely, biochars B1 and B3 were not completely removed. Particle size distribution of CB1 and CB3 had a much larger spectrum with high amounts of micron size particles (data not shown). Additionally, Spokas and colleagues showed the physical disintegration of biochar in the presence of water resulting in biochar micro- and nanoparticles with the same chemical structure (approximated by O:C ratios) as the original biochar particles [26]. These particles also seem to contribute to the slow release of nitrate.

### Nitrate extraction after compost sterilization

One batch of composts was γ-sterilized before the extraction to check if microbial ammonification and nitrification during the extraction (up to 167 h at 23°C) might cause slow release of nitrate. However, there were no considerable differences compared to the extraction of non-γ-irradiated samples (Fig 2C). This was expected, as the activity of a soil or compost microbial community should be very low under the hypersaline conditions (2 M KCl = 149.1 g L^-1^ salt) during extraction. Thus, slow release of nitrate is not a biotic process.

### H_2_O_2_ as extractant

In order to test if nitrate was retained in a matrix of labile organic carbon, we combined 2 M KCl with 3% H_2_O_2_ solution during the first step of extraction. H_2_O_2_ induces abiotic oxidative mineralization of organic matter [27]. If nitrate was retained e.g. by an organic coating on the co-composted biochar particles [28, 29], oxidative mineralization could accelerate the rate of nitrate release by removing this organic matrix. Except for CB2, extraction with H_2_O_2_-containing extractant resulted in increased total release of NO_3_^-^ for all composts (Fig 2D), probably due to the mineralization and abiotic oxidation of compost organic nitrogen [27]. The elevated content of iron in B2 (Table 1, 130 g kg^-1^) might have protected the biochar-amended compost CB2 from oxidation, as Fe(III) is known to catalyze the decomposition of H_2_O_2_ [30]. The amount of slowly released NO_3_^-^ increased for CB1, but was not affected for CB2 and slightly decreased for CB3.

The increase for CB1 mainly happened during the second extraction, which is remarkably as the second extraction was not relevant during all other extractions (12 mg N kg^-1^ average release of NO_3_^-^ during second extractions for all extractions after 12–14 months, here: 87 mg N kg^-1^). Residual H_2_O_2_, e.g. retained by biochar B1, might have still reacted with the compost organic matter. However, it is not clear why this happened only in CB1.

Decreased amounts of slow released NO_3_^-^ in CB3 show that captured nitrate on biochar B3 became partly accessible by the oxidative mineralization by H_2_O_2_ and thus was already extracted with the first step. This indicates that B1 and B3 may have different mechanisms to capture NO_3_^-^. Organic coating of co-composted biochar [28, 29] might contribute to the nitrate capture of B3 as an additional reservoir for nitrate.

### Nitrate extraction from soil

**Fig 3:**
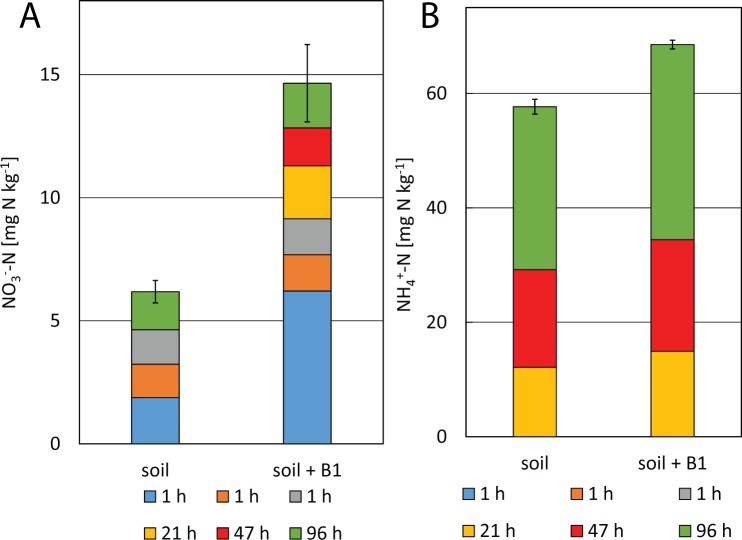
Mineral nitrogen release from soil without and with biochar. (A) Nitrate and (B) ammonium released from soil and biochar-(B1-type)-amended soil. Error bars indicate standard errors of triplicate extractions summed for 6 subsequent extractions. T-test revealed that the total amounts of extracted nitrate are significantly different (p = 0.0071), unlike the amounts of ammonium (p = 0.067) (B). The N content of some extracts was below detection limit, thus these sections are not visible in the tacked bar graphs.

Unlike compost, the sandy loam soil showed a certain amount of slow release nitrate (Fig 3A) which comprised 70% of the total extracted nitrate. According to standard protocols, nitrate should be completely extractable with water or 2 M KCl within 1 h of incubation [13]. These protocols assume that nitrate moves freely within leachate in soils with predominantly negative surface charges [31] Nitrate retention has so far mainly been discussed in the context of highly weathered tropic soils with predominantly positive charged surfaces [31]. However, the soil used in this study was a temperate soil. The clay fraction of the soil, iron minerals or soil organic matter might be responsible for the slow release. Komarneni and colleagues developed a nitrate slow release fertilizer based on anionic clays that completely released NO_3_^-^ only after 3 to 7 days of extraction with an artificial soil solution [32]. Also Bhardwaj and colleagues produced a slow release fertilizer based on clay and zeolite [33]. However, nitrate retention and soil clay content do not necessarily correlate [34]. Klučáková showed that nitrate can sorb to humic acids by so far unknown mechanisms [35]. However, it is unknown how this effects the extractability of nitrate from soil. Klučáková used humic acids extracted from lignite. Additionally, the control compost used in this study, i.e. a material with very high organic matter content, did not show significant nitrate retention. Also other soil constituents have been suggested to sorb nitrate, including iron minerals and allophane [34]. Further studies comparing soils with different clay content, different types of clays, and different content of iron and organic matter are necessary to elucidate what mechanisms might be responsible for the retention of nitrate in non-biochar amended soil.

The amendment with 60 Mg ha^-1^ biochar 3 years before the sampling for this study slightly decreased the relative amount of nitrate extracted during extractions steps 2 to 6 (58%). However, biochar amendment significantly (t-test, p = 0.007) increased the amount of total extractable nitrate by a factor of 2, although both treatments initially received the same amount of fertilizer and were managed identically over three years.

### Release of ammonium

Considerable quantities of NH_4_^+^ have been released in all extractions (Figs 1C, 1D, 2E–2H and 3B). For both compost and soil extractions, the quantities of released ammonium rather seemed to depend on the duration of extraction than on the presence or absence of biochar. Elongated extractions with aqueous solutions have been used previously to estimate the amount of soil or compost organic N that can be mineralized. Cordovil and colleagues found that hot (100°C) extraction with 2 M KCl for 4 h could estimate the N mineralization from organic amendments during 4 weeks of incubation in soil [36]. Curtin and Campbell suggested “anaerobic incubation” of soil in distilled water (1:10) for 1 week at 40°C to estimate mineralizable nitrogen from subsequent NH_4_^+^-N quantification [37]. In our experiments, all extractions showed that biochar had limited impact on the quantity of mineralizable N with CB2 having apparently the lowest mineralization rate. The highest quantities of NH_4_^+^ during the first step of extraction were found in γ-sterilized compost due to cell lysis, the subsequent abiotic ammonification and the absence of nitrifying bacteria. Still, ongoing ammonification during the extraction even after γ-sterilization strongly suggests an abiotic nature of this process.

### Potential impact of biochar on the fate of N during composting

Elemental analysis (Table 2) showed that total N (N_tot_) of the composts is one order of magnitude higher than the total amount of extracted mineral N, i.e. the sum of NO_3_^-^ and NH_4_^+^ (NO_2_^-^ was negligible for all measurements). Biochar-amended composts CB2 and CB3 have the same N content as the non-biochar-amended control, although the initial N content before the composting was lower due to the “dilution” of the N rich manure by the 4.3% amendment with the biochar. CB1, however, had a lower total N content compared to all other substrates. Taking into account that the biochar amended composts had 4.3% less manure-N in the beginning, this indicates that biochar B1 could not reduce losses of nitrogen (leaching, gaseous losses) compared to the control compost, while B2 and B3 seem to improve the preservation of feedstock N. However, because the composting was performed in mechanically aerated windrow at sub-industrial scale, it was not possible to assess a holistic mass balance with C and N budgets. Thus, our conclusions on total nitrogen losses during the composting process have to be interpreted with care. We monitored pH during the composting process, as pH controls the losses of ammonia during composting (data not shown). However, we did not observe considerable differences.

**Table 2:** Mineral and total N content of non-amended control and biochar-amended composts.

		Control	CB1	CB2	CB3
Mineral N [NO_3_^-^] + [NH_4_^+^], [g kg^-1^]	6 months	0.8±0.1	0.6±0.0	0.5±0.0	0.8±0.1
	6 months, 70°C	1.1±0.1	0.8±0.0	0.7±0.0	1.3±0.2
				
	12 months	1.2±0.0	1.1±0.1	1.0±0.0	1.1±0.1
	12 months w/o biochar	1.2±0.1	1.1±0.0	1.0±0.1	1.2±0.1
	12 months sterilized	1.9±0.1	1.8±0.1	1.6±0.1	2.0±0.1
	12 months H_2_O_2_	1.5±0.2	1.3±0.1	1.0±0.0	1.5±0.0
				
Total N [g kg^-1^]	elemental analysis	15.4±0.6	13.9±0.1	15.6±0.7	15.8±0.0

Amount of mineral N (= [NO_3_^-^] + [NH_4_^+^]) quantified in repeated extractions after 6 and 12 months. Amount of total N as quantified with elemental analysis of freeze dried compost.

### Agroecological relevance and future research

In this study we showed that biochar nitrate capture is a relevant process controlling total nitrate budgets of biochar-amended composts and soils. Even biochar micro- or nano-particles seemed to contribute to the nitrate capture and the resulting slow release of nitrate. Nitrate slow release was based on abiotic mechanisms because it was still observable after γ-sterilization of the composts. Non-biochar-amended compost did not show slow release of nitrate. Thus, biochar was the only component in a biochar-amended manure compost that caused nitrate capture and enabled its slow release. Soil, in our case a sandy loam, however, could retain nitrate beyond the conventional 1 h 2 M KCl extraction as well, because 70% of total extracted nitrate (6.2 mg N kg^-1^) was only released during subsequent extractions, too. However, biochar did not further increase the relative contribution of slow released nitrate to the total pool of extractable nitrate (70% slow release in soil vs. 58% slow release in biochar amended soil), but increased the total pool of extractable nitrate in soil (14.6 mg N kg^-1^).

Increasing nitrate retention in agroecosystems is critical. Galloway and colleagues argue that the global production of reactive nitrogen species, which are predominately used as fertilizers, increased by 120% since 1970 due to a “pervasive inefficiency” [38] that promotes the formation of greenhouse gas nitrous oxide in agricultural soils [39]. Additionally, leached nitrate can be looked at as unintended fertilization of adjacent ecosystems and thus can lead to eutrophication and decrease the diversity of plant species e.g. in natural grasslands [40]. Ultimately, leached nitrate can be transported to the ocean and can cause eutrophication and the formation of hypoxic zones [41]. Overall, the anthropogenic alteration of global nitrogen cycling is considered the most urgent thread to maintaining the Earth in a resilient and habitable state [42]. Inefficient use of nitrogen fertilizers resulting in NO_3_^-^ leaching to the groundwater is a major contributor to this situation [43]. Future research on biochar nitrate capture and slow release needs to aim at understanding the mechanisms of this so far hardly understood retention of nitrate. This might create the scientific basis for both a new generation of slow release fertilizers that also reduce nitrate leaching from agroecosystems on the long run. We suggest three complementary research strategies:

First, **soil nitrate capture** should generally be investigated in more detail. Our study showed that nitrate capture and slow release is not an exclusive characteristic of biochar, but can be an intrinsic feature of soils, too. However, it is unknown which soil constituents are responsible for this effect. So called “abiotic nitrate incorporation” into soil organic matter has been suggested in earlier studies [44–46]. Different types of clays have been successfully tested as basic constituents in slow release fertilizers [32, 47]. Thus, clays and biochars should be compared in sorption and desorption experiments to gain further mechanistic insights. Such experiments have been already performed with respect to N leaching in amended soils but not for slow release fertilizer development [14].

Second, **kinetics of biochar slow release of nitrate** [48] should be quantified for contrasting biochars and after nitrate sorption under contrasting conditions (pristine biochar vs. aged biochar, different nitrate sources, etc.). While a broad range of studies on the sorption of nitrate to biochar [49, 50] or on the effect of biochar on nitrate leaching from soil columns [51–53], have been performed, only few studies also focused on desorption of nitrate from biochar after sorption. Release kinetics under different conditions can reveal insights into strategies to maximize biochar nitrate capture, and the trade-off between plant availability and loss through leaching. For this purpose, it will be necessary to understand the underlying mechanisms on the molecular scale. New methods to quantify the amount of captured nitrate more rapidly could aid in this research. This study showed that the addition of 3% H_2_O_2_ to 2 M KCl or the increase of temperature during extraction (70°C) did not uniformly accelerate the extraction of captured nitrate for all biochars, but revealed new mechanistic insights. Kammann and colleagues [11] used electro-ultrafiltration to gain mechanistic insights into the release characteristics. However, the method only released a fraction <50% of the nitrate that could potentially be released. This was confirmed by methodical investigations of Haider and colleagues [10]. Thus electro-ultrafiltration offers mechanistical insights but no means for quick and complete extraction of captured N from biochar particles. More methodical studies will be needed.

Third, **release of organic N and C from (aged) biochar, biochar amended composts and soil** should be quantified alongside with nitrate release. Organic N contributes to plant nutrition; Kammann and colleagues showed that co-composted biochar slowly released organic N in addition to low amounts of NH_4_^+^, and dominantly NO_3_^-^ [11]. They also showed an exponential rise-to-maximum correlation of the release of nitrate to the release of dissolved organic carbon with R^2^ values of >0.99 that argue for a mechanistic physico-chemical relationship between nitrate and DOC [11]. The present study showed that H_2_O_2_ reduced the amount of slowly released nitrate at least for one biochar-amended compost (CB3), indicating a contribution of labile organic matter to nitrate capture, e.g. by an organic coating. Organic coatings on co-composted biochar particles have been proposed previously [28, 29] and might serve as an additional reservoir for nitrate.

The proposed research agenda could contribute to a more holistic understanding or biochar nitrate capture and slow release, which may facilitate the development of biochar-based slow release fertilizers. This will contribute to reducing the environmental impact of fertilization. Promoting the use of biochar as fertilizer carrier for anion retention in soils will contribute to the protection of our water bodies. So far, the widespread application of biochar in agriculture is considered desirable mainly due to its climate change mitigation potential [54–57] and the hope to mimic the historic examples of extremely fertile anthropogenic soils [6, 58]. However, overall yield increase by biochar is just mediocre (+18%), while “high yield increases are more of an exception than the rule” as stated by Jeffery and colleagues [7].

Recent research suggests that biochar needs to be “loaded” with nutrients by e.g. co-composting [11], or by macerating in urine [9], or by co-application with mineral fertilizer directly in the rhizosphere [8] to result into remarkable yield increase. Thus, understanding the interaction of biochar and nutrients, particularly the mobile anion nitrate, is vital to achieve biochar-mediated growth promotion and thus provide an economic incentive for farmers to use biochar. Research on biochar nitrate capture mechanisms will provide the basic knowledge to develop slow release fertilizers into commercial products for routine application in sustainable agriculture.

Future studies on biochar and nitrogen transformations should always consider biochar nitrate capture as a potential pool of nitrogen, when data based on extraction are evaluated. Standard extraction procedures might underestimate the extractable nitrate content of biochar amended soils and fertilizers.

## Supporting information

S1 File Quantification of mineral N species in the presence of DOC. Introduction and results and discussion of a study on the potential impact of dissolved organic carbon (DOC) on the quantification of nitrate, nitrite and ammonium.
(PDF)
