# Supplementary material for: Nitrate capture and slow release in biochar amended compost and soil
Source: PLoS One. 2017 Feb 15;12(2):e0171214. doi: 10.1371/journal.pone.0171214 (PMC5310885; doi:10.1371/journal.pone.0171214)
Supplement: S1 File — Introduction and results and discussion of a study on the potential impact of dissolved organic carbon (DOC) on the quantification of nitrate, nitrite and ammonium. (PDF) [file pone.0171214.s001.pdf]

Nitrate capture and slow release in biochar amended compost and soil

Short Title: Biochar nitrate capture in compost and soil

Supporting information: Quantification of mineral N species in the presence of DOC

Nikolas Hagemann[1*], Claudia I. Kammann[2], Hans-Peter Schmidt[3], Andreas Kappler[1], and Sebastian Behrens[4,5*]

[1] Geomicrobiology, Center for Applied Geoscience, University of Tuebingen, Sigwartstrasse 10, 72076 Tuebingen, Germany

[2] Working group Climate Change Research for Special Crops, Department for Soil Science and Plant Nutrition, Hochschule Geisenheim University, Von-Lade-Str. 1, Geisenheim D-65366, Germany

[3] Ithaka Institute for Carbon Strategies, Ancienne Eglise 9, Arbaz 1974, Switzerland

[4] Department for Civil, Environmental, and Geo-Engineering, University of Minnesota, 500 Pillsbury Drive S.E., Minneapolis, MN 55455-0116, United States

[5] BioTechonology Institute, 140 Gortner Labs, 1479 Gortner Avenue, St. Paul, Mn 55108-6106, United States

*: Corresponding Authors: nikolas@hagemann.at (NH), sbehrens@umn.edu (SB)

Introduction

It was previously reported that dissolved organic carbon (DOC) and iron can interfere with colorimetric quantification of nitrate (1). This is relevant in environmental samples such as aqueous extracts obtained from soil or compost, but was also shown to have an overall limited effect (2). To assess the potential impact of DOC and native iron concentrations of compost on colorimetric nitrate quantification on a continuous flow analysis (SEAL Analytical, Germany), we used aqueous compost extracts with 5, 25 and 100 mg L^{-1} DOC and spiked them with different concentrations of nitrite, nitrate and ammonium.

Results and Discussion

We found that nitrite was quantified accurately throughout all experimental conditions (Tab. S1-S3).

We found that nitrate was quantified accurately in the presence of 5 and 25 mg L^{-1} DOC. Nitrate quantification in the presence of 100 mg L^{-1} DOC could not be evaluated due to the high background concentration of nitrate in the compost extract. As extracts obtained from compost need to be diluted for measurements, 100 mg L^{-1} DOC was not a relevant concentration for obtaining the data presented in the manuscript. We found that nitrate was quantified accurately in the presence of both 5-25 mg L^{-1} DOC and 1 mg L^{-1} NO_2^--N. However, the presence of 5 mg L^{-1} NO_2^--N resulted in an overestimation of nitrate of up to 13%. However, extracts of mature compost barely contain measurable amounts of nitrite, i.e. this effect is not relevant to this study.

Ammonium was quantified accurately in the presence of 5 mg L^{-1} DOC, but underestimated by up to ~10% in the presence of 25-100 mg L^{-1} DOC.

Collectively, there is no evidence that the conclusions of this study that are based on the colorimetric quantification of nitrate, nitrite and ammonium are related to, or biased by, experimental artefacts. Nitrate and nitrite were quantified accurately under relevant conditions. Concentrations of ammonium might have been underestimated, however, this would not affect the overall results and conclusions.

	NO_2^- added [mg L^{-1}]	NO_3^- added [mg L^{-1}]	NH_4^+ added [mg L^{-1}]	values obtained from Seal Software				NO_2^- expected [mg L^{-1}]	NO_3^- expected [mg L^{-1}]	NH4 expected [mg L]	Δ NO_2^- [mg L^{-1}]	Δ NO_2^- [% of expected]	Δ NO_3^- [mg L^{-1}]	Δ NO_3^- [% of expected]	Δ NH_4^+ [mg L^{-1}]	Δ NH_4^+ [% of expected]
				NO_3^-+NO_2^- [mg N L^{-1}]	NO_2^- [mg N L^{-1}]	NH_4^+ [mg N L^{-1}]	NO_3^- (calc) [mg N L^{-1}]									
**Nitrite, Ammonia**				0.991	0.008	0.108	0.99	0.01	1.01	0.11	0.00	-14%	-0.02	-1%	0.00	-2%
				0.992	0.008	0.105	0.99	0.01	1.01	0.11	0.00		-0.02		0.00	
	0.50		0.50	1.485	0.495	0.586	1	0.51	1.01	0.61	-0.01	-3%	-0.01	0%	-0.02	-4%
	0.50		0.50	1.495	0.497	0.581	1	0.51	1.01	0.61	-0.01		-0.01		-0.03	
	1.00		1.00	2.008	0.995	1.056	1.02	1.01	1.01	1.11	-0.01	-1%	0.01	1%	-0.05	-5%
	1.00		1.00	2.003	1.003	1.061	1.01	1.01	1.01	1.11	-0.01		0.00		-0.05	
	2.00		2.00	2.977	1.986	2.012	1	2.01	1.01	2.11	-0.02	-1%	-0.01	0%	-0.10	-4%
	2.00		2.00	3.001	2.004	2.031	1	2.01	1.01	2.11	-0.01		-0.01		-0.08	
	5.00		5.00	6.112	5.058	5.079	1.06	5.01	1.01	5.11	0.05	1%	0.05	7%	-0.03	-1%
	5.00		5.00	6.142	5.047	5.078	1.1	5.01	1.01	5.11	0.04		0.095		-0.03	
**Nitrate**				1.05	0.008	0.11	1.05	0.01	1.01	0.11	0.00	14%	0.04	1%	0.00	2%
				0.998	0.013	0.111	0.99	0.01	1.01	0.11	0.00		-0.02		0.00	
		0.50		1.462	0.011	0.106	1.46	0.01	1.51	0.11	0.00	14%	-0.05	-2%	0.00	-3%
		0.50		1.472	0.01	0.105	1.48	0.01	1.51	0.11	0.00		-0.03		0.00	
		1.00		1.969	0.009	0.104	1.98	0.01	2.01	0.11	0.00	3%	-0.02	-1%	0.00	-5%
		1.00		1.983	0.01	0.103	1.99	0.01	2.01	0.11	0.00		-0.01		-0.01	
		2.00		2.948	0.008	0.101	2.97	0.01	3.01	0.11	0.00	-8%	-0.03	-1%	-0.01	-6%
		2.00		2.923	0.009	0.103	2.95	0.01	3.01	0.11	0.00		-0.05		-0.01	
		5.00		6.018	0.009	0.102	6.08	0.01	6.01	0.11	0.00	-8%	0.075	2%	-0.01	8%
		5.00		6.073	0.008	0.132	6.14	0.01	6.01	0.11	0.00		0.135		0.02	
**Nitrate in Presence of Nitrite**	1.00		1.00	2.008	1	1.065	1.02	1.01	1.01	1.11	-0.01	0%	0.01	1%	-0.04	-4%
	5.00		5.00	6.184	5.064	5.104	1.13	5.01	1.01	5.11	0.05		0.13	12%	0.00	0%
	1.00	0.50	1.00	2.525	1.003	1.068	1.54	1.01	1.51	1.11	-0.01	0%	0.03	2%	-0.04	-4%
	5.00	0.50	5.00	6.752	5.079	5.104	1.7	5.01	1.51	5.11	0.07		0.20	13%	0.00	0%
	1.00	1.00	1.00	3.04	1.004	1.07	2.06	1.01	2.01	1.11	-0.01	0%	0.06	3%	-0.04	-3%
	5.00	1.00	5.00	7.248	5.07	5.11	2.21	5.01	2.01	5.11	0.06		0.21	10%	0.00	0%
	1.00	2.00	1.00	4.003	1.007	1.074	3.04	1.01	3.01	1.11	0.00	1%	0.04	1%	-0.03	-3%
	5.00	2.00	5.00	8.315	5.085	5.124	3.28	5.01	3.01	5.11	0.08		0.28	9%	0.02	0%
	1.00	5.00	1.00	7.123	1.002	1.067	6.22	1.01	6.01	1.11	-0.01	0%	0.22	4%	-0.04	-4%
	5.00	5.00	5.00	11.287	5.089	5.116	6.3	5.01	6.01	5.11	0.08		0.30	5%	0.01	0%

Tab. S1: Nitrate, nitrate and ammonium spike experiment with 5 mg L^{-1} DOC. Addition of 5 mg NO_2^--N is highlighted in blue. Expected concentrations are calculated as the sum of N added and the background concentrations measured when 0 N was added. Differences (Δ) in N concentrations expressed in [%] of the expected concentration are highlighted in yellow, if the differences are ~10% or higher and the measured values are within the range of calibration (0.25 – 7.5 mg N L^{-1}).

	NO_2^- added [mg L^{-1}]	NO_3^- added [mg L^{-1}]	NH_4^+ added [mg L^{-1}]	values obtained from Seal Software NO_3^-+NO_2^- [mg N L^{-1}]	NO_2^- [mg N L^{-1}]	NH_4^+ [mg N L^{-1}]	NO_3^- (calc) [mg N L^{-1}]	NO_2^- expected [mg L^{-1}]	NO_3^- expected [mg L^{-1}]	NH4 expected [mg L]	Δ NO_2^- [mg L^{-1}]	Δ NO_2^- [% of expected]	Δ NO_3^- [mg L^{-1}]	Δ NO_3^- [% of expected]	Δ NH_4^+ [mg L^{-1}]	Δ NH_4^+ [% of expected]
**Nitrite, Ammonia**				5.16	0.007	0.293	5.24	0.01	5.26	0.16	0.00	2%	-0.02	0%	0.14	29%
				5.177	0.014	0.111	5.25	0.01	5.26	0.16	0.00		-0.01		-0.05	
	0.50		0.50	5.646	0.499	0.584	5.23	0.51	5.26	0.66	-0.01	-2%	-0.03	0%	-0.07	-11%
	0.50		0.50	5.732	0.501	0.588	5.32	0.51	5.26	0.66	-0.01		0.06		-0.07	
	1.00		1.00	6.191	1.004	1.069	5.28	1.01	5.26	1.16	-0.01	-1%	0.02	0%	-0.09	-8%
	1.00		1.00	6.189	0.998	1.06	5.28	1.01	5.26	1.16	-0.01		0.02		-0.10	
	2.00		2.00	7.221	1.982	2.014	5.33	2.01	5.26	2.16	-0.03	-2%	0.07	2%	-0.14	-7%
	2.00		2.00	7.257	1.977	2.014	5.38	2.01	5.26	2.16	-0.03		0.12		-0.14	
	5.00		5.00	10.751	5.101	5.13	5.76	5.01	5.26	5.16	0.09	2%	0.5	10%	-0.03	-1%
	5.00		5.00	10.747	5.095	5.125	5.76	5.01	5.26	5.16	0.08		0.5		-0.03	
**Nitrate**				5.191	0.007	0.112	5.29	0.01	5.26	0.16	0.00	-2%	0.03	0%	-0.04	-29%
				5.17	0.013	0.111	5.26	0.01	5.26	0.16	0.00		0		-0.05	
		0.50		5.688	0.01	0.108	5.79	0.01	5.76	0.16	0.00	-7%	0.03	1%	-0.05	-31%
		0.50		5.733	0.009	0.108	5.84	0.01	5.76	0.16	0.00		0.08		-0.05	
		1.00		6.172	0.008	0.107	6.29	0.01	6.26	0.16	0.00	-22%	0.03	1%	-0.05	-31%
		1.00		6.201	0.008	0.109	6.33	0.01	6.26	0.16	0.00		0.07		-0.05	
		2.00		7.234	0.008	0.091	7.39	0.01	7.26	0.16	0.00	-17%	0.13	2%	-0.07	-46%
		2.00		7.281	0.009	0.079	7.44	0.01	7.26	0.16	0.00		0.18		-0.08	
		5.00		10.64	0.008	0.067	10.88	0.01	10.26	0.16	0.00	-22%	0.62	6%	-0.09	-61%
		5.00		10.576	0.008	0.055	10.82	0.01	10.26	0.16	0.00		0.56		-0.10	
**Nitrate in Presence of Nitrite**	1.00	0.00	1.00	6.315	0.997	1.004	5.45	1.01	5.26	1.16	-0.01	0%	0.19	4%	-0.15	-13%
	5.00	0.00	5.00	10.761	5.109	5.089	5.79	5.01	5.26	5.16	0.10		0.53	10%	-0.07	-1%
	0.50	0.50	0.50	6.274	0.493	0.515	5.92	0.51	5.76	0.66	-0.02	-2%	0.16	3%	-0.14	-22%
	1.00	0.50	1.00	6.788	1.008	0.985	5.92	1.01	5.76	1.16	0.00		0.16	3%	-0.17	-15%
	5.00	0.50	5.00	11.077	5.139	5.098	6.09	5.01	5.76	5.16	0.13	0%	0.33	6%	-0.06	-1%
	0.50	1.00	0.50	6.846	0.499	0.491	6.51	0.51	6.26	0.66	-0.01		0.25	4%	-0.17	-25%
	1.00	2.00	1.00	8.537	1.006	0.957	7.73	1.01	7.26	1.16	0.00	1%	0.47	6%	-0.20	-17%
	5.00	2.00	5.00	11.163	5.151	5.033	6.17	5.01	7.26	5.16	0.14		-1.09	-15%	-0.12	-2%
	1.00	1.00	1.00	7.403	1.07	0.937	6.5	1.01	6.26	1.16	0.06	4%	0.24	4%	-0.22	-19%
	5.00	1.00	5.00	11.171	5.138	5.028	6.2	5.01	6.26	5.16	0.13		-0.06	-1%	-0.13	-2%

Tab. S2 Nitrate, nitrate and ammonium spike experiment with 25 mg L^{-1} DOC. Addition of 5 mg NO_2^--N is highlighted in blue. Expected concentrations are calculated as the sum of N added and the background concentrations measured when 0 N was added. Differences (Δ) in N concentrations expressed in [%] of the expected concentration are highlighted in yellow, if the differences are ~10% or higher and the measured values are within the range of calibration (0.25 – 7.5 mg N L^{-1}).

	NO_2^- added [mg L^{-1}]	NO_3^- added [mg L^{-1}]	NH_4^+ added [mg L^{-1}]	values obtained from Seal Software				NO_2^- expected [mg L^{-1}]	NH4 expected [mg L]	Δ NO_2^- [mg L^{-1}]	Δ NO_2^- [% of expected]	Δ NH_4^+ [mg L^{-1}]	Δ NH_4^+ [% of expected]
				NO_3^-+NO_2^- [mg N L^{-1}]	NO_2^- [mg N L^{-1}]	NH_4^+ [mg N L^{-1}]	NO_3^- (calc) [mg N L^{-1}]						
**Nitrite, Ammonia**	0.00		0.00	11.649	0.01	-0.06	11.96	0.01	-0.07	0.00	0%	0.01	0%
	0.00		0.00	11.635	0.016	-0.07	11.94	0.01	-0.07	0.00		-0.01	
	0.50		0.50	11.649	0.491	0.387	11.47	0.51	0.44	-0.02	-4%	-0.05	-12%
	0.50		0.50	11.639	0.493	0.378	11.46	0.51	0.44	-0.02		-0.06	
	1.00		1.00	11.547	0.989	0.843	10.86	1.01	0.94	-0.02	-2%	-0.09	-10%
	1.00		1.00	11.559	0.987	0.831	10.88	1.01	0.94	-0.03		-0.10	
	2.00		2.00	11.554	1.983	2.129	9.85	2.01	1.94	-0.03	-2%	0.19	10%
	2.00		2.00	11.653	1.982	2.135	9.96	2.01	1.94	-0.03		0.20	
	5.00		5.00	11.796	5.077	5.168	6.92	5.01	4.94	0.06	1%	0.23	5%
	5.00		5.00	11.82	5.081	5.217	6.95	5.01	4.94	0.07		0.28	

Tab. S3 Nitrate, nitrate and ammonium spike experiment with 100 mg L^{-1} DOC. Differences (Δ) in N concentrations expressed in [%] of the expected concentration are highlighted in yellow, if the differences are ~10% or higher and the measured values are within the range of calibration (0.25 – 7.5 mg N L^{-1})

Experimental

A compost extract was obtained by shaking non-biochar amended compost (Con) 1:10 in de-ionized water for 72 h. The extract was filtered to 1 µm to avoid clogging of the continuous flow analysis.

We used a 50 mg N L^{-1} standard solutions for nitrite, nitrate and ammonium to spike the extracts to increase concentrations in the final sample by 0.5-5 mg N L^{-1}. Final samples had a volume of 1 mL, contained 5, 25 or 100 mg L^{-1} DOC and contained 2 M KCl. The experiment was conducted in duplicate.

Nitrate, nitrite and ammonium were quantified by continuous flow analysis (SEAL Analytical, Germany) as described in the main paper.

Acknowledgements

We thank Ellen Röhm for support during this experiment.

References

1. Colman BP, Fierer N, Schimel JP. Abiotic nitrate incorporation in soil: is it real? Biogeochemistry. 2007;84(2):161-9.

2. Davidson EA, Dail DB, Chorover J. Iron interference in the quantification of nitrate in soil extracts and its effect on hypothesized abiotic immobilization of nitrate. Biogeochemistry. 2008;90(1):65-73.
